# Complete Genome Sequence of the Hyperthermophilic and Piezophilic Archeon *Thermococcus piezophilus* CDGS^T^, Able To Grow under Extreme Hydrostatic Pressures

**DOI:** 10.1128/genomeA.00610-16

**Published:** 2016-07-14

**Authors:** Cécile Dalmasso, Philippe Oger, Damien Courtine, Myriam Georges, Ken Takai, Lois Maignien, Karine Alain

**Affiliations:** a Université de Bretagne Occidentale (UBO, UBL), Institut Universitaire Européen de la Mer (IUEM)–UMR 6197, Laboratoire de Microbiologie des Environnements Extrêmes (LM2E), Plouzané, France; b CNRS, IUEM–UMR 6197, Laboratoire de Microbiologie des Environnements Extrêmes (LM2E), Plouzané, France; c Ifremer, UMR 6197, Laboratoire de Microbiologie des Environnements Extrêmes (LM2E), Technopôle Pointe du Diable, Plouzané, France; d Université de Lyon, INSA Lyon, CNRS UMR 5240, Villeurbanne, France; e Department of Subsurface Geobiological Analysis and Research (D-SUGAR), Japan Agency for Marine-Earth Science and Technology (JAMSTEC), Yokosuka, Japan

## Abstract

We report the genome sequence of *Thermococcus superprofundus* strain CDGS^T^, a new piezophilic and hyperthermophilic member of the order *Thermococcales* isolated from the world’s deepest hydrothermal vents, at the Mid-Cayman Rise. The genome is consistent with a heterotrophic, anaerobic, and piezophilic lifestyle.

## GENOME ANNOUNCEMENT

*Thermococcus piezophilus* strain CDGS^T^ was isolated from the deep-sea hydrothermal vent field Beebe, in the Cayman Trough (1–3), at 4964-m water depth (18°32.7881′ N, 81°43.11844′ W) (4). It can grow at temperatures ranging from 60 to 90°C (optimum: 75°C) and optimally under 50 MPa. This strain holds the current record of pressure range for growth, growing effectively from atmospheric pressure to at least 120 MPa, and with difficulty up to 130 MPa (4).

Whole-genome shotgun sequencing was carried out using a combination of IonTorrent (318 Chip, HiQ chemistry; IGFL, ENS, Lyon, France) and PacBio (1 large insert library + 2 smartcells; Duke University, Durham, USA) technologies. *De novo* assembly of the PacBio reads was obtained using the HGAP assembler included in a local installation of the PacBio SMRT portal (v2.3.0). The complete genome sequence was then corrected by mapping assembly of the IonTorrent reads on the PacBio contig using MIRA 4 and the Newbler 2.8 suite of programs, and then manually curated. The hybrid data assembly consists of one single chromosome of 1,928,919 bp, with an average G+C content of 51.11%, and does not possess any extrachromosomal elements. A total of 2,418 coding DNA sequences (CDSs) were identified with the MaGe platform (5–7), as well as one copy of the 16S-23S operon, two copies of 5S rRNA, 45 tRNA genes, and 15 miscellaneous RNA. Additionally, the genome contains one integrase, one transposase and clustered regularly interspaced short palindromic repeat (CRISPR) loci associated with *cas* genes (*cas*1, *cas*2, *cas*3, *cas*4, *cas*5, *cas*6, *csm*2), suggesting that this strain has a certain genomic plasticity and suffered rearrangements.

This strain is most closely related to *Thermococcus onnurineus* NA1 (8), as indicated by *in silico* DNA-DNA hybridization of their genomes (predicted value = 50.50% using the genome-to-genome distance calculator GGDC2.0 [9–11]). However, despite the highly conserved synteny between these two genomes, a few genomic inversions were observed, as well as differences in gene content and gene nature. The core genome is composed of only 1,297 CDS. Both strains possess the metabolic pathways for organotrophic growth on peptides, amino acids, or sugars. Notably, *T. piezophilus* possesses the full glycolysis V pathway, the d-mannose degradation pathway, the glycerol degradation pathway, and the degradation pathways for several amino acids (asparagine, aspartate, citrulline, homocystéine, glycine). It possesses also the polysulfide respiration pathway. In contrast to *T. onnurineus*, its genome does not encode all the proteins (from the transporters to the cleavage enzymes) necessary to grow by carboxydotrophy or by formate oxidation.

Consistent with its piezophilic lifestyle, *T. piezophilus* possesses several complete pathways for the synthesis of compatible solutes (glutamine, glutamate, glycine, myo-inositol, and N-acetylglucosamine). Additionally, it harbors several hydrogenase gene clusters, including hydrogenases and sulfhydrogenases, known to be regulated by pressure in other piezophilic *Thermococcus* species (12).

### Nucleotide sequence accession number.

The genome sequence has been deposited in GenBank under the accession number CP015520.
