# Erratum for Dalmasso et al., Complete Genome Sequence of the Hyperthermophilic and Piezophilic Archaeon *Thermococcus piezophilus* CDGS^T^, Able To Grow under Extreme Hydrostatic Pressures

**DOI:** 10.1128/genomeA.01018-16

**Published:** 2016-09-15

**Authors:** Cécile Dalmasso, Philippe Oger, Damien Courtine, Myriam Georges, Ken Takai, Loïs Maignien, Karine Alain

**Affiliations:** a Université de Bretagne Occidentale (UBO, UBL), Institut Universitaire Européen de la Mer (IUEM)–UMR 6197, Laboratoire de Microbiologie des Environnements Extrêmes (LM2E), Plouzané, France; b CNRS, IUEM–UMR 6197, Laboratoire de Microbiologie des Environnements Extrêmes (LM2E), Plouzané, France; c Ifremer, UMR 6197, Laboratoire de Microbiologie des Environnements Extrêmes (LM2E), Technopôle Pointe du Diable, Plouzané, France; d Université de Lyon, INSA Lyon, CNRS UMR 5240, Villeurbanne, France; e Department of Subsurface Geobiological Analysis and Research (D-SUGAR), Japan Agency for Marine-Earth Science and Technology (JAMSTEC), Yokosuka, Japan

## ERRATUM

Volume 4, no. 4, e00610-16, 2016. Page 1: The article title should read as given above, and the organism name in the first sentence of the abstract should be given as *Thermococcus piezophilus* strain CDGS^T^.

Page 2: Reference 4 should read as follows.

4. **Dalmasso C, Oger P, Selva G, Courtine D, L’Haridon S, Garlaschelli A, Roussel E, Miyazaki J, Reveillaud J, Jebbar M, Takai K, Maignien L, Alain K.** *Thermococcus piezophilus* sp. nov., a novel hyperthermophilic and piezophilic archaeon with a broad pressure range for growth, isolated from a deep hydrothermal vent at the Mid-Cayman Rise. Syst Appl Microbiol, in press.

